# Markers of Local Inflammation and Bone Resorption in the Acute Diabetic Charcot Foot

**DOI:** 10.1155/2018/5647981

**Published:** 2018-08-02

**Authors:** Rasmus Bo Jansen, Tomas Møller Christensen, Jens Bülow, Lene Rørdam, Niklas Rye Jørgensen, Ole Lander Svendsen

**Affiliations:** ^1^Copenhagen Diabetes Foot Center (CODIF), Bispebjerg Hospital, University of Copenhagen, 2400 Copenhagen NV, Denmark; ^2^Department of Endocrinology, Bispebjerg Hospital, University of Copenhagen, 2400 Copenhagen NV, Denmark; ^3^Department of Clinical Physiology and Nuclear Medicine, Bispebjerg Hospital, University of Copenhagen, 2400 Copenhagen NV, Denmark; ^4^Department of Clinical Biochemistry, Rigshospitalet, University of Copenhagen, 2600 Glostrup, Denmark; ^5^Odense Patient data Explorative Network (OPEN), Odense University Hospital/Institute of Clinical Research, University of Southern Denmark, Odense, Denmark

## Abstract

**Objective:**

Due to the localized nature of Charcot foot, systemically altered levels of inflammation markers can be difficult to measure. The aim of this study was to investigate whether it is possible to detect an arteriovenous (A-V) flux in any locally produced inflammatory biomarkers from an acute Charcot foot by comparing local and systemic measurements.

**Methods:**

We included patients with acute diabetic Charcot foot. Blood was sampled from the vena saphena magna on the distal part of the crus bilaterally as well as from the arteria radialis. To minimize the A-V shunting effect, the feet were externally cooled with ice water prior to resampling.

**Results:**

Both before and after cooling, the A-V flux of interleukin-6 (IL-6) between the Charcot feet and the arterial level was significantly higher than the flux between the healthy feet and the arterial level (Δvalue_before_: 7.25 versus 0.41 pg/mL, resp., *p* = 0.008; Δvalue_after_: 10.04 versus 1.68 pg/mL, resp., *p* = 0.032). There were no differences in the fluxes for other markers of inflammation.

**Conclusion:**

We have found an increased A-V flux of IL-6 in the acute diabetic Charcot foot compared to the healthy foot in the same patients.

## 1. Introduction

Charcot osteoarthropathy is a rare disorder manifesting with aseptic inflammation and hyperemia in and around load-bearing bones and tissues. The process is normally unilateral and leads to progressive, uncontrolled resorption and degeneration of bone mass, resulting in spontaneous fatigue bone fractures [1–4]. While different locations have been described, the most common is in the feet (Charcot foot (CF)) [5, 6], where the process can cause deformity, ulcerations, and amputations. The Charcot inflammation can be located at different sites in the affected foot, most prominently in the midfoot [2, 7].

The precise pathological mechanisms underlying Charcot foot are still not fully understood. However, it is dependent on relatively unimpaired lower limb blood flow and established peripheral neuropathy [8–11]. It can be triggered by a number of diseases, although today most cases occur in individuals with diabetes mellitus [1, 2, 12].

Recent evidence suggests that the initial inflammation is provoked by repeated local microtrauma and dysregulated bone resorption [13–16], which in turn initiates the inflammatory process.

Several studies have reported changes in biomarkers of bone resorption and inflammation in individuals with Charcot foot [17–24], and it seems that the inflammation leads to microstructural changes in the affected bones [25, 26].

Related to this, studies have explored the possible relationship between interleukin levels and acute Charcot foot [17, 27–29] and found increased levels of interleukin-1 receptor antagonist (IL-1RA), tumor necrosis factor *α* (TNF-*α*), interleukin-6 (IL-6), and interleukin-17 subtypes A, E, and F (IL-17A/E/F), as well as decreased levels of interleukin-1*β* (IL-1*β*) and interleukin-8 (IL-8).

In addition, many individuals with Charcot foot also seem to have a degree of vascular calcification and inflammation [30, 31]. This is of particular interest due to the connection between vascular calcification, neuropathy, and the nuclear factor-*κ*B (NF-*κ*B) system, as described by Petrova and Shanahan [32]. A possible way to assess this could be through the system of advanced glycation end products (AGEs) and their soluble receptors (sRAGE) [33–35].

However, the biomarkers in question might only be produced locally around the inflamed bones in the foot, which means that the signal on a systemic level can be difficult to register. Furthermore, a general systemic release of these biomarkers might happen in response to a number of inflammatory processes unrelated to the Charcot foot.

Therefore, it is plausible that a stronger and more specific signal from an acute Charcot foot might be achieved by measuring the flux of a specific marker between the local venous concentration in the foot and the arterial concentration. Local sampling from the dorsal venous arch of the foot in acute Charcot feet has previously been done by Gough et al. and Pearson et al. [21, 23], although neither measured all the markers discussed here. To our knowledge, local fluxes of inflammatory biomarkers across an acute Charcot foot have not been measured previously.

The aim of this study was to investigate whether it is possible to detect a flux in any locally produced biomarkers from an acute Charcot foot by measuring the arteriovenous (A-V) difference.

## 2. Materials and Methods

### 2.1. Participants

We included participants with acute Charcot foot, recruited at the Copenhagen Wound Healing Center at Bispebjerg Hospital, Denmark, and at the Steno Diabetes Center, Gentofte, Denmark. The participants were referred by specialists after thorough physical examination, full blood panel, X-ray, bone scintigraphy, and/or MRI. All participants were examined as close as possible to the reported outbreak of the acute Charcot symptom (<3 months).

Exclusion criteria included no diabetes mellitus, temperature difference < 2°C between the feet, duration > 3 months, foot ulcers, prior foot surgery, new objective foot deformities, bilateral Charcot foot, infection in the foot, antiosteoporotic medication, arterial insufficiency, or foot or toe amputation on either side.

To confirm that the Charcot foot still had a high activity on the day of examination as assessed by a locally elevated blood flow, this was measured in the feet with venous occlusion plethysmography [36]. Foot temperature and foot somatosensoric neuropathy as assessed by biothesiometry were measured on the study day as well.

The study was approved by the Regional Ethical Committee for Copenhagen.

### 2.2. Arteriovenous Flux

To measure the fluxes in biomarker production in the acute Charcot foot, blood was sampled from the vena saphena magna on the distal part of the crus above the ankle. This was done on both the affected (Charcot) side and the healthy side. Arterial blood was sampled from the a. radialis (or from the a. brachialis if the a. radialis was inaccessible).

The venous drainage of the foot happens primarily through the veins saphena magna and parva, while the deep veins only play a minor role. The saphena veins connect through the dorsal venous arch on the dorsal side of the foot. The dorsal venous arch collects blood from both superficial and deep veins in the foot, as well as from the networks rete venosus plantare and rete venosus dorsale pedis. The superficial and deep veins of the foot are linked by communicating perforant veins. The few valves present in these perforants are turned so that blood can only run from the deep to the superficial veins, thus helping with thermoregulation and pressure absorption. This means that parts of the drainage of the deep foot happen through the superficial veins, which can thus be sampled from a superficial vein on the lower leg [37–39].

A portion of the blood flow in the feet bypasses normal microcirculatory exchange by shunting directly through A-V anastomoses. This is in part a thermoregulatory effect and is thus more prevalent at higher skin temperatures [40]. As the shunted blood will not be exposed to any biomarkers produced in the deep foot tissue, this A-V shunting in effect dilutes the signal of any inflammatory biomarkers in a mixed venous sample. To minimize this shunting effect in our setup, we cooled down the feet externally with cold water prior to the final sampling (*t*_ice_).

### 2.3. Experimental Setup

**Figure 1:**
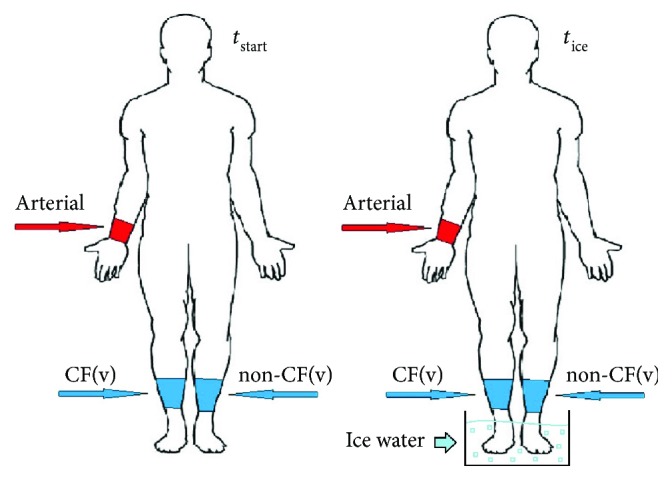
Sites of arterial (red) or venous (blue) blood samples before (*t*_start_) and after (*t*_ice_) external cooling of the feet. Arterial samples were taken from the a. radialis, or from the a. brachialis if the a. radialis was inaccessible. Venous samples were taken from a large superficial vein at the third distal part of the crus both on the side with a Charcot foot (CF(v)) and on the side without a Charcot foot (non-CF(v)).

All three sites were sampled simultaneously (*t*_start_) (Figure 1). Fluxes between the arterial and venous concentrations were calculated as vein − artery to get a positive gradient if the Charcot foot produced the biomarker in question. After sampling, both feet were cooled down for approximately 10 minutes in an icy water bath, while foot temperature was measured. The three sites were then sampled again while the participants kept their feet in the water (*t*_ice_).

In the following, venous samples from the acute Charcot foot are denoted as CF(v) and venous samples from the non-Charcot foot are denoted as non-CF(v).

The blood samples were centrifuged at 4°C and stored at −80°C. All samples were analyzed together at the end of the study.

### 2.4. Biomarkers

To estimate the existing interleukin profile and the highest relative concentrations, *t*_start_ samples were analyzed on a Bio-Plex System multiplex immunoassay screening panel (Bio-Rad Laboratories Inc., 4000 Alfred Nobel Drive, Hercules, California 94547, USA). The panel used screened for IL-1*β*, IL-1RA, IL-6, IL-8, IL-17A, and TNF-*α*, with the best signals detected for IL-6 and IL-8.

All analyses were performed by Biolab, Department of Clinical Biochemistry, Rigshospitalet, University of Copenhagen, Denmark. Special ELISA setups were used for IL-6, IL-8, free soluble receptor activator of nuclear factor kappa-*Β* ligand (fsRANK-L), osteoprotegerin (OPG), IL-17F, sRAGE, and AGEs. The remaining samples were analyzed as part of the daily hospital sample routines. The accepted intraindividual sample CV for all assays was 20%.

- AGEs were measured with a Human sandwich ELISA AGE kit (Nordic BioSite AB, Propellervägen 4A, 183 62 Täby, Sweden) (kit serial number LS-F10641-1; range 0.78–50 ng/mL).
- IL-6 was measured with an IL-6 Quantikine HS ELISA kit (Bio-Techne Ltd., 614 McKinley Place NE, Minneapolis, MN 55413, USA) (kit serial number HS600B).
- IL-8 was measured with a Human CXCL8 Quantikine kit (Bio-Techne Ltd., 614 McKinley Place NE, Minneapolis, MN 55413, USA) (kit serial number D8000C).
- IL-17F was measured with a Human IL-17F Platinum ELISA kit (AH-Diagnostics A/S, Runetoften 18, DK-8210 Aarhus V, Denmark) (kit serial number BMS2037/2).
- Assays for fsRANK-L, osteoprotegerin, and sRAGE were performed as previously described [41].

### 2.5. Statistical Analysis

Data are presented as mean ± 1 SD or range unless otherwise noted. An *α*-level of <0.05 was considered significant. Normal distribution in data was tested using Shapiro-Wilks tests. No transformations were used. *t*-tests or paired *t*-tests were used for variance analysis between groups in normally distributed data sets. For data sets not normally distributed, nonparametric tests in the form of the Mann-Whitney rank-sum test were used, while Wilcoxon signed-rank tests were used for comparing paired samples.

Statistics and general data handling were done using IBM SPSS Statistics v. 23 by IBM Corporation, SIGMAPLOT v. 11.0.0.77 by Systat Software Inc., Microsoft Excel 2000 v. 9.0.2812 by Microsoft Corporation, and Apache OpenOffice 4.0.1 by the Apache Software Foundation.

## 3. Results

### 3.1. Participants

We included 5 patients with acute Charcot foot. In total, 22 patients were screened for inclusion. Of these, 7 patients were excluded due to having foot ulcers or receiving foot surgery and/or ulcer debridement before the examinations. Another 7 did not want to participate or were unable to participate due to personal reasons, while 3 patients had had their Charcot foot for too long to be considered acute (duration > 3 months).

The average time from the reported onset of symptoms to examination was 7.2 weeks. The Charcot feet were on average 2.6°C warmer than the contralateral and had a 3 times increased blood flow. All 5 patients had recently started off-loading treatment with an AirCast® removable walker boot before measurements. All 5 patients were diagnosed with stage 0 Charcot foot.

Anthropometric data for the participants are listed in Table 1, along with the results for biothesiometry, venous occlusion plethysmography, and arterial samples of markers of bone health taken prior to the cooling of the feet (*t*_start_). There were a significant higher temperature and blood flow in the acute Charcot feet compared to the healthy feet.

### 3.2. Multiplex Data

For IL-1*β*, IL-1RA, IL-17A, and TNF-*α*, almost all measured values were below the multiplex limit of detection in all samples. This was tested with both serum and plasma. The detection limits were 1.32 pg/mL for IL-1, 29.64 pg/mL for IL-1RA, 7.99 pg/mL for IL-17A, and 12.72 pg/mL for TNF-*α*. There was a single signal in one patient in IL-1RA and IL-17A (not the same patient for both markers).

The average level of IL-6 detected was 10.6 pg/mL, and for IL-8, it was 12.5 pg/mL.

### 3.3. Measurements before Cooling

Measurements from all three sites (arterial, CF(v), and non-CF(v)) before and after cooling are listed in Table 2. At *t*_start_, there were no differences in the levels of fsRANK-L, OPG, IL-6, IL-8, sRAGE, or AGEs—neither between arterial and CF(v) nor between CF(v) and non-CF(v) samples. The highest relative numerical difference was for IL-6 in arterial versus CF(v) levels (7.31 versus 14.56) (*p* = 0.109). It was not possible to measure IL-17F as it was below the assay detection limit of 7.8 pg/mL for all samples at *t*_start_ and *t*_ice_.

The venous-arterial flux of IL-6 between the Charcot feet and the arterial level was significantly higher than the flux between the healthy feet and the arterial level (Δvalues: 7.25 versus 0.41 pg/mL, resp.) (*p* = 0.008). There were no differences in fsRANK-L, OPG, IL-8, sRAGE, or AGEs.

### 3.4. Measurements after External Cooling

The ice bath used for cooling maintained an average temperature of 7.7°C, and it was used for cooling for an average of 11.6 min. The ice bath cooled the Charcot feet at an average of 11.0°C (from 33.7 to 22.7°C), and the non-Charcot feet were cooled at an average of 12.9°C (from 31.1 to 18.2°C). Temperatures in each foot before and after cooling are listed in Table 3.

**Figure 2:**
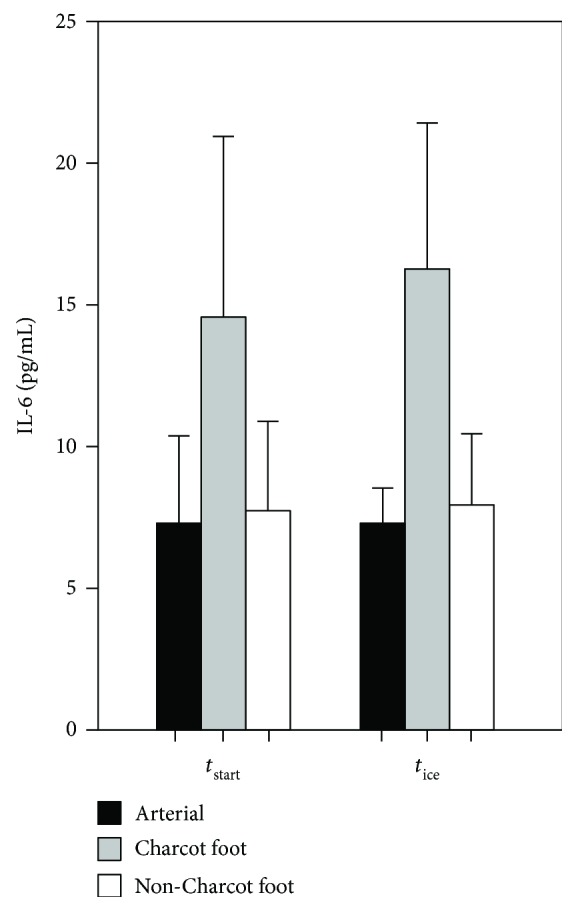
Levels of interleukin 6 (IL-6) in arterial and local venous samples in both feet (CF(v) and non-CF(v)) before (*t*_start_) and after (*t*_ice_) external cooling with ice water. Bars = mean; error bars = SEM.

At *t*_ice_, there was a significantly elevated level of IL-6 (Δvalue: 10.04 pg/mL) in the Charcot feet compared to the arterial value (*p* = 0.049) (Figure 2). There was also a significantly elevated level of AGEs (Δvalue: 2.5 ng/mL) (*p* = 0.002) (Table 2). There were no differences in fsRANK-L, OPG, IL-8, or sRAGE.

The venous-arterial flux for IL-6 at *t*_ice_ was still significantly increased in the Charcot feet (CF(v)-arterial) compared to the healthy feet (non-CF(v)-arterial) (Δvalues: 10.04 versus 1.68 pg/mL) (*p* = 0.032). There were no differences in the fluxes for fsRANK-L, OPG, IL-8, sRAGE, or AGEs.

The fsRANK-L/OPG ratio at *t*_ice_ was 3.7 in the arterial sample, 4.0 in the CF(v) sample, and 3.8 in the non-CF(v) sample and did not differ in a one-way ANOVA on ranks (*p* = 0.970).

## 4. Discussion

In this study, we have tested a novel approach to evaluating the local inflammatory activity in an acute Charcot foot by measuring the venous-arterial flux across the Charcot foot while lowering the possible dilution from A-V shunting by externally cooling the foot.

The data show a difference in the venous-arterial flux of IL-6 both before and after external cooling. We also saw a two-fold elevated level of IL-6 in the Charcot foot compared to the arterial level after cooling, indicating a local production of IL-6. It is interesting that both IL-6 and AGEs only show a significant difference between the Charcot foot and arterial level after cooling, thus possibly indicating an effect of limiting A-V shunting in the feet before sampling.

The results are mostly in line with what other groups have found. Divyateja et al. indicated an increased median IL-6 level in the Charcot foot [24], while both Petrova et al. [17] and Folestad et al. [29] have suggested increased levels of IL-6 systemically (although Folestad et al. did not find an initially increased level of IL-6).

Unlike Folestad et al., we have been unable to demonstrate high levels of IL-17F systemically in acute Charcot patients [27]. However, they did show an initial low level of IL-17F (corresponding to the time where we performed our sampling), and additionally, they used high-sensitivity ECL as opposed to the ELISA that we used.

A finding of locally increased levels of IL-6 is of particular interest for several reasons. As a proinflammatory cytokine, its presence supports the theory regarding the pathogenesis of acute Charcot foot as put forth by, for instance, Jeffcoate et al. [13]. Furthermore, IL-6 is involved in bone resorption through osteoclastic differentiation and activation [42–45]. Thus, the finding further supports local osteoclastic hyperactivation as a central element in the Charcot foot bone metabolism and confirms the findings of IL-6 in osteoclasts in bone samples from Charcot feet as seen by Baumhauer et al. [46]. The source of this local production of IL-6 remains unknown.

Recently, Petrova et al. reported that OPG was elevated in patients with Charcot foot without a corresponding elevation in RANK-L [17] and that osteoclasts from patients with Charcot foot can be modulated by TNF-*α* through RANK-L [47]. It is important to note however that elevated OPG levels could be associated with neuropathy in itself [48].

Ndip et al. have indicated that individuals with Charcot foot have an increased RANK-L/OPG ratio and suggest that this could play a role in medial vascular calcification [22]. We have also previously shown a higher RANK-L/OPG ratio in patients with acute Charcot foot than non-Charcot diabetic controls [41]. In the current setup, we did not find a difference in the venous-arterial flux or a locally elevated RANK-L/OPG ratio. However, this was not to be expected either as both markers only circulate in very small quantities and furthermore have half-lives sufficient to recirculate the vascular system many times, making it difficult to detect a local difference.

Regarding the increased level of AGEs after cooling, it is unclear whether this is an expression of a local increase in production of AGEs due to cooling, a by-product of the Charcot inflammation, or merely a random sampling variation.

The presence of tissue-bound receptors for AGEs (RAGE) has been associated with impaired bone matrix mineralization and enhanced osteoclast formation [49]. AGEs have been linked to a negative modification of collagen integrity and fragile bones in general [50–52]. Thus, if there is indeed an increased level of AGEs present in Charcot feet, this might account for a further weakening of the bones. Furthermore, there is a link between increased levels of RAGE and activation of the NF-*κ*B system and several associated cytokines [53, 54]. As such, it shares a common pathway of influence of osteoclastic activation with RANK-L/OPG and by extension IL-6.

### 4.1. Strengths and Limitations

To our knowledge, this is the first time that the local venous-arterial flux across an acute Charcot foot has been studied. Furthermore, we are unaware of other studies that have limited the local A-V shunting effect prior to measuring a Charcot foot.

The study was limited by the number of available participants. In total, we screened 22 patients and most of these were excluded due to foot ulcers or extended time from the symptom onset to diagnosis. Thus, part of the recruitment issue was the rigorous exclusion criteria needed to ensure that any possible findings were not clouded by infections, surgery, or prolonged Charcot inflammation.

Furthermore, most of the assays we have used have a limited accuracy and substantial intraindividual variations, and thus it was difficult to register any possible differences. These variations might be the reason why we saw an increase in AGEs after cooling in the Charcot foot compared to the arterial level. Unless more accurate assays are developed, future tests in a similar setup could be performed with multiple samples from each site and each time point to help alleviate this issue.

## 5. Conclusion

In conclusion, we have found an increased venous-arterial flux of IL-6 in the acute diabetic Charcot foot compared to the healthy foot. We also found an increased level of IL-6 and AGEs in the acute Charcot foot compared to the arterial level after, but not before, externally cooling the feet.

## Figures and Tables

**(a):** 

	Data listed as mean; range or *n*
Age (years)	48.6; 26.0
Sex (m/f)	3/2
Affected foot (left/right)	1/4
Diabetes type (I/II)	2/3
Diabetes duration (years)	19.2; 31.0
HbA1c (mmol/mol) (31–44 mmol/mol)	73; 53
Ca^2+^(free, ionized) (mmol/L) (1.18–1.32 mmol/L)	1.24; 0.14
PTH (pmol/L) (1.6–6.9 pmol/L)	4.5; 4.3
CRP (mg/L) (<10 mg/L)	9.8; 15.0
25-OH-vitamin D (nmol/L) (50–160 nmol/L)	36.7; 52.6
Alkaline phosphatase (bone specific) (*μ*g/L) (<20 *μ*g/L)^$^	20.3; 7.3
CTX (ng/L) (<630 ng/L)^$^	240; 0.5
P1NP (*μ*g/L) (22–87 *μ*g/L)^$^	48.3; 53.8
Osteocalcin (*μ*g/L) (9–42 *μ*g/L)	25.3; 42.1

**(b):** 

	Charcot foot	Contralateral foot	Difference, *p* value
Foot temperature (CF/non-CF) (°C)	33.7	31.1	Δ2.6, *p* = 0.004^∗^
Biothesiometry (CF/non-CF) (V)	42	39	Δ3, *p* = 0.648
Plethysmography (CF/non-CF) (mL/(100 g·min))	6.9	1.8	Δ5.1, *p* = 0.045^∗^

^∗^Significant at the chosen *α*-level of 0.05. ^$^Reference range listed for 50 y.o. male where ranges differ with age and/or sex.

**Table 2:** Levels of inflammation markers in local venous samples in the acute Charcot foot (CF(v)), the healthy foot (non-CF(v)), and arterial samples from the a. radialis. Measurements listed before (*t*_start_) and after (*t*_ice_) external cooling of both feet with ice water.

	Sampling site	*t*_start_	*t*_ice_
fsRANK-L (pmol/L)	Arterial	0.14 ± 0.12	0.13 ± 0.11
	CF(v)	0.13 ± 0.11	0.14 ± 0.11
	non-CF(v)	0.13 ± 0.11	0.14 ± 0.13

OPG (pmol/L)	Arterial	6.5 ± 5.4	6.6 ± 5.1
	CF(v)	6.4 ± 5.8	7.3 ± 5.9
	non-CF(v)	6.3 ± 5.5	7.5 ± 6.1

IL-6 (pg/mL)	Arterial	7.31 ± 6.88	6.25 ± 5.21
	CF(v)	14.56 ± 14.27	16.29 ± 11.45
	non-CF(v)	7.71 ± 7.07	7.93 ± 5.70

IL-8 (pg/mL)	Arterial	15.6 ± 7.9	13.1 ± 6.3
	CF(v)	13.4 ± 4.4	12.1 ± 6.3
	non-CF(v)	14.5 ± 9.6	11.5 ± 4.7

sRAGE (ng/L)	Arterial	845 ± 266	860 ± 247
	CF(v)	833 ± 292	878 ± 298
	non-CF(v)	827 ± 252	911 ± 293

AGEs (ng/mL)	Arterial	6.2 ± 7.7	5.4 ± 7.4
	CF(v)	5.7 ± 6.8	7.9 ± 7.1
	non-CF(v)	5.9 ± 7.2	8.4 ± 8.4

Data listed as mean ± 1 SD. fsRANKL = free soluble receptor activator of nuclear factor-*κ*B; OPG = osteoprotegerin; IL-6/IL-8 = interleukin 6/interleukin 8; sRAGE = soluble receptor for advanced glycation end products; AGEs = advanced glycation end products.

**Table 3:** Temperature measurements on the feet of each individual patient during the study day.

Patient number	Charcot foot temperature (°C)		Non-Charcot foot temperature (°C)	
	Before cooling (*t*_start_)	After cooling (*t*_ice_)	Before cooling (*t*_start_)	After cooling (*t*_ice_)
1	32.0	20.0	29.7	18.0
2	33.8	25.9	31.7	18.6
3	33.7	20.2	32.3	20.6
4	34.8	21.8	31.0	17.0
5	34.4	25.7	30.7	17.0

## Data Availability

All data, in anonymised form, are available upon contact to the corresponding author.

## Supplementary Materials

Supplementary Materials Appendix 1 contains the primary output from the multiplex assay. The strength of the responses was used to evaluate the most likely markers for the final assay panel.
