# Supplementary material for: Markers of Local Inflammation and Bone Resorption in the Acute Diabetic Charcot Foot
Source: J Diabetes Res. 2018 Aug 2;2018:5647981. doi: 10.1155/2018/5647981 (PMC6098852; doi:10.1155/2018/5647981)
Supplement: Supplementary Materials — Appendix 1 contains the primary output from the multiplex assay. The strength of the responses was used to evaluate the most likely markers for the final assay panel. [file 5647981.f1.doc]

**Appendix 1:**

Below are listed the primary output of the Multiplex analyses, upon which we, in part, based the final assay range for assessment.
